# A Frequency Signature RFID Chipless Tag for Wearable Applications

**DOI:** 10.3390/s19030494

**Published:** 2019-01-25

**Authors:** Laura Corchia, Giuseppina Monti, Luciano Tarricone

**Affiliations:** Department of Engineering for Innovation, University of Salento, via per Monteroni, 73100 Lecce, Italy; laura.corchia@unisalento.it (L.C.); luciano.tarricone@unisalento.it (L.T.)

**Keywords:** chipless tag, fully-textile, RFID, wearable

## Abstract

In this paper, a frequency-signature Radio-Frequency Identification (RFID) chipless tag for wearable applications is presented. The results achieved for a fully-textile solution guaranteeing a seamless integration in clothes are reported and discussed. The proposed tag consists of two planar monopole antennas and a 50 Ω microstrip line loaded with multiple resonators. In order to achieve a compact size, the resonators are slotted on the ground plane of the microstrip line. As for the antennas, the same geometry was exploited for both the TX and the RX tag antenna. In particular, it consists of a proximity fed planar monopole on a ground plane. The selected geometry guarantees easy integration with the multi-resonator structure. Numerical and experimental data referring to a 2-bit implementation are presented and discussed. For fabricating all the prototypes, a layer of pile was used as a substrate, while an adhesive non-woven conductive fabric was exploited for the fabrication of the conductive parts. Experimental tests demonstrate that although the performance of the final device strongly depends on the properties of the used materials and on the imperfections of the fabrication process, the proposed frequency-signature RFID chipless tag is suitable for wearable applications, such as anti-counterfeiting systems and laundry labels.

## 1. Introduction

So far, the success of wearable electronics has been hampered by technological limits and, in particular, by the lack of materials and manufacturing techniques that would enable a seamless integration of the electronic parts in the garments. In fact, although several conductive fabrics are available on the market [1,2,3] and different low-cost fabrication techniques have been proposed in literature [3,4,5,6,7,8,9,10,11,12,13,14,15], the wearability of smart clothes and their robustness in operations such as washing, drying and ironing are still strongly limited by the use of chips and ICs requiring tin soldering [5,6]. This problem is overcome by chipless devices, such as chipless Radio Frequency Identification (RFID) tags [16,17,18,19,20,21,22,23,24,25,26,27,28,29,30,31,32,33,34]. 

The use of chipless tags guarantees important practical advantages, such as seamless integration with the clothes, real-time operations, potentially infinite “service-life”, low environmental impact, and low cost [16,17,18]. Furthermore, chipless tags are able to work in harsh scenarios where the correct operation of conventional RFID technology could be compromised by the exposure of ICs to high temperatures or hazardous environments [16]. On the other hand, encoding data without the presence of ICs represents a major challenge. For this reason, industrial and academic efforts are mainly focused on the investigation of new approaches guaranteeing efficient encoding [16]. 

More recently, the chipless RFID technology has also been exploited for the fabrication of sensors [16,18,23,25,26,27,28]. In this regard, numerous chipless sensors have been proposed in the literature, but for the majority of these devices, reliability and reproducibility are still critical issues [18].

**Figure 1:**
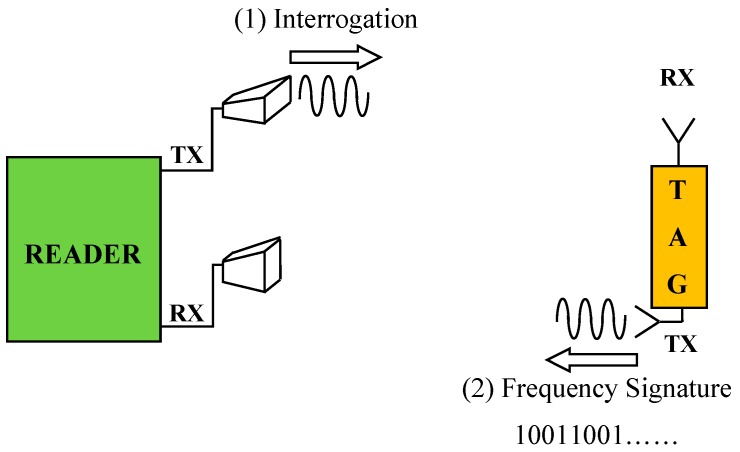
Schematic representation of a generic multi-resonator chipless tag Radio Frequency Identification (RFID) system [19].

In terms of the approach adopted for encoding data, the following classification can be used for RFID chipless tags: spectral signature [19], phase encoding [29], polarization diversity [30], and time domain signature [31]. However, aside from the adopted approach, the operating mechanism is the same: there is a reader that sends an interrogation signal to the transponders; the transponders reflect back a signal encapsulating the encoded data (see Figure 1). 

As for wearable applications, both simple chipless tags and sensors have been proposed in the literature [23,24,32,33,34]. Among these, in [23], the results achieved for a sewn chipless RFID sensor tag are presented. Numerical and experimental results reported in the paper demonstrate the correct operation of the chipless tag in the frequency range, 3–6 GHz. In more detail, the sewn stretchable sensor consists of a rectangular loop sewn on to a stretchable fabric. A conductive thread is used for fabricating the loop. The achieved results confirm that the proposed device is suitable to be used as a strain sensor.

In Reference [24], an RFID chipless tag for people identification is presented. The proposed device consists of L-shaped scatterers able to depolarize the incident wave and to generate a cross-polarized signal. The capability of depolarizing the interrogation signal allows for a robust decoding even when the tag is placed in direct contact with the human body. However, numerical and experimental results demonstrate that the performance of the proposed tag is strongly affected by bending. In particular, a wrong ID decoding occurs if the tag is placed on a curved region of the human body.

In Reference [33], the authors have proposed a fully textile frequency-signature chipless tag consisting of a microstrip line loaded with multiple resonators. Each resonator corresponds to a single bit. Numerical and experimental results achieved for a 3-bit prototype have demonstrated the suitability of the proposed device for RFID wearable applications requiring a low number of bits, such as anti-counterfeiting systems and laundry labels [23]. In this paper, new numerical and experimental results are reported and discussed. It is shown that the proposed device is a low-cost solution which allows a seamless integration in garments.

The paper is structured as follows: in Section 2, details on the geometry and numerical results achieved for both the single components (adjustable multi-stopband structure and monopole antennas) and the tag connected with antennas are given, experimental results are reported in Section 3, and finally, a brief discussion and some conclusions are reported in Section 4.

## 2. Geometry and Numerical Results

The proposed wearable frequency-signature chipless tag consists of a 50 Ω microstrip line loaded with compact resonators and two wideband antennas. In particular, the microstrip line acts as a stopband structure. 

As shown in Figure 1, a generic RFID system based on a multi-resonator tag also comprises a reader equipped with two antennas. The operating principle is very simple: (1) the reader sends an interrogation signal to the tag and (2) the tag replies, sending a backscattered signal whose frequency spectrum contains the encoded data. Both the reader and the transponder antennas are cross-polarized in order to minimize possible interferences between the interrogation signal and the backscattered encoded signal [19]. In this paper, the attention is focused on the tag system.

In the following subsections, details on the geometry and numerical results achieved for both the single components (adjustable multi-stopband structure and wideband antennas) and the overall tag system will be given. 

### 2.1. Multi-Resonator Structure

**Figure 2:**
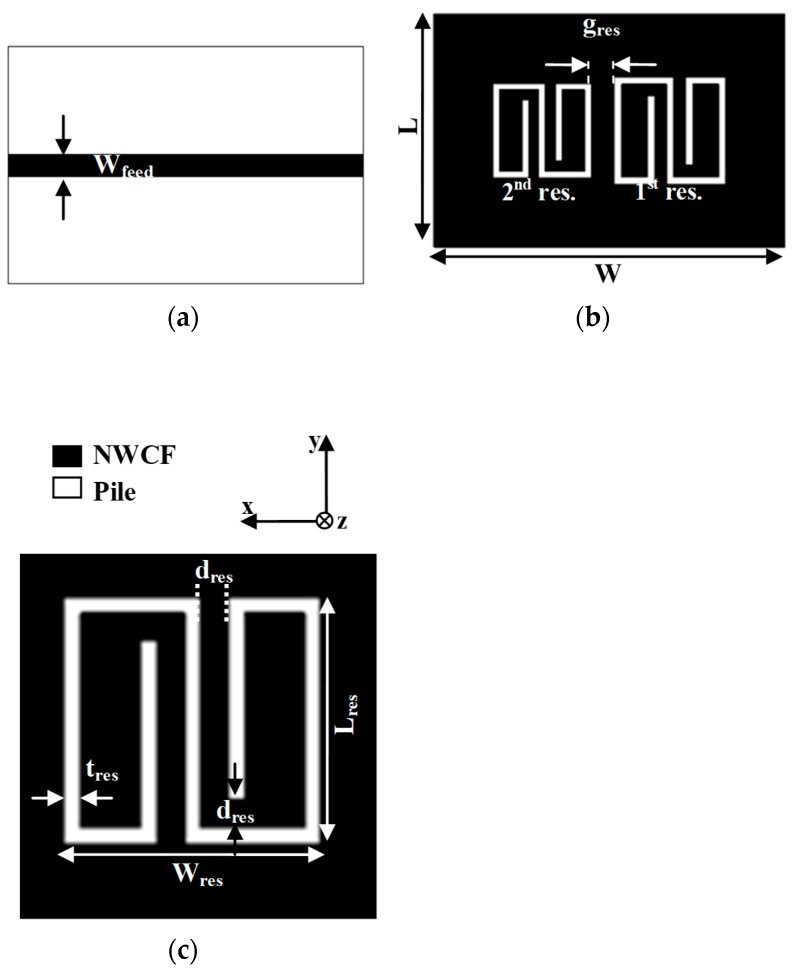
Geometry of the proposed multi-stopband structure: (**a**) front view of a 2-bit multi-stopband structure; (**b**) back view of a 2-bit multi-stopband structure; (**c**) geometry of the single resonator.

The proposed multi-resonator structure is illustrated in Figure 2. The resonators are slotted on the ground plane of the microstrip line. The front view and the back view of a 2-bit multi-stopband structure are shown in Figure 2a,b, respectively; the single resonator is illustrated in Figure 2c.

**Figure 3:**
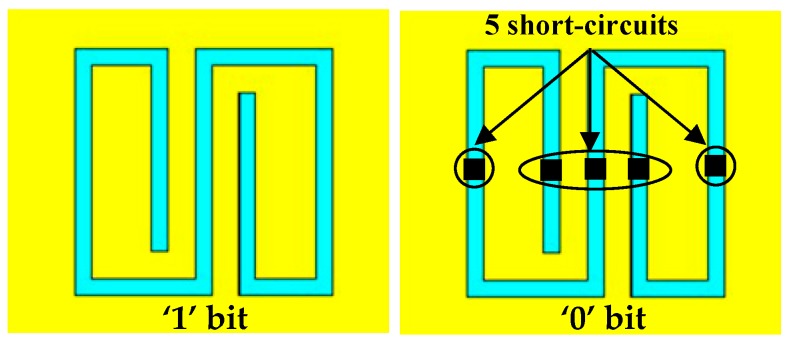
To encode a 0 bit, five short-circuits are used to modify the layout of the single resonator.

The design of the multi-stopband structure was performed with the commercial full-wave simulator CST Microwave Studio [35]. The design process consisted of two main steps: 1) first, the resonator operating at the lowest frequency was optimized; 2) second, the resonators operating at higher frequencies were obtained through a uniform scaling of the first resonator on the x–y plane. The adopted approach can be summarized through the following equation, which describes the relationship between the total area of the first resonator and the total area of the n^th^ resonator:

(1) $$A_{n}=A_{1}\cdot {\left({\mathrm{SF}}_{n}\right)}^{2},\;n>1$$

where A_1_ is the area of the first resonator (on the x–y plane), A_n_ is the area of the n^th^-resonator (on the x–y plane), and SF_n_ is the n^th^ scale factor. Each resonator introduces a transmission zero (i.e., a relative minimum of the transmission coefficient of the microstrip line), whose central frequency is controlled by the parameter SF_n_. Consequently, each resonator corresponds to a single bit and the presence of a minimum amplitude (and a phase variation) of the S_21_ parameter represents a logic 1; conversely, the absence is encoded as a “0” bit. To encode a logic 0, the layout of the corresponding resonator is modified as illustrated in Figure 3.

The frequency shift between two consecutive resonant peaks depends on the scale factor and, obviously, for a given bandwidth, higher scale factors allow a higher number of bits to be encoded; on the other hand, excessively high scale factors lead to a complex decoding.

**Figure 4:**
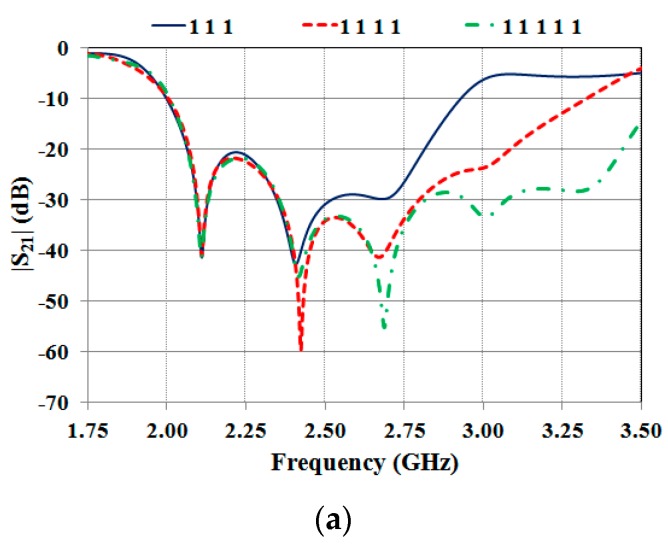

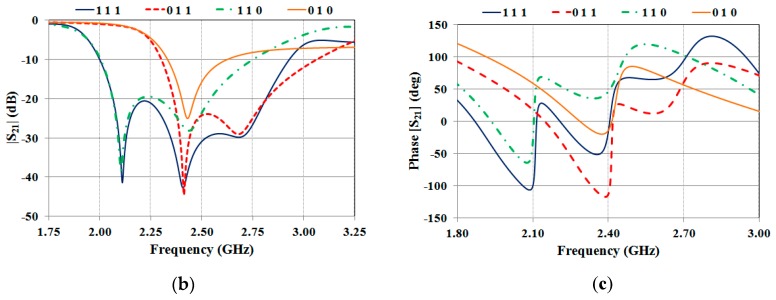
Numerical results achieved for different multi-resonator structures: (**a**) amplitude of the S_21_ achieved for multi-stopband structures with 3, 4, and 5 bits; amplitude (**b**) and phase (**c**) of the S_21_ calculated from full-wave simulations for different configurations of a 3-bit structure.

A comparison between numerical results achieved for multi-resonator structures having a different number of bits is illustrated in Figure 4a. 

Numerical results obtained for four different configurations of a 3-bit tag are reported in Figure 4b,c. These results demonstrate that the data encapsulated in the frequency spectrum can be decoded by analyzing both the amplitude (Figure 4a,b) and the phase (Figure 4c) of the S_21_ parameter.

As it can be seen from Figure 4b,c, for the different configurations, the position of the minima of the |S_21_| and of the phase variations of the S_21_ are both stable in frequency. This means that when the geometry of one or more resonators is modified to achieve a logic 0, the resonances corresponding to the other resonators are not significantly influenced.

### 2.2. TX and RX tag Antennas

**Figure 5:**
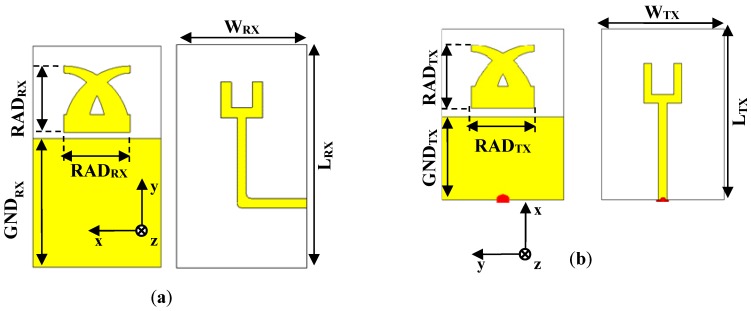
Geometries and dimensions of the wideband antennas proposed as RX tag antenna (**a**) and TX tag antenna (**b**).

As shown in Figure 1, a multi-resonator chipless tag also includes TX and RX antennas. Hence, to complete the geometry of the proposed device, two planar monopole antennas were also designed. The geometry adopted for the TX and the RX antennas, which is illustrated in Figure 5, was proposed for the first time in [36]. Each antenna is a proximity-fed planar monopole on a ground plane, optimized to operate in the frequency range 2–4 GHz. This solution guarantees a compact size and simplifies the integration with the multi-stopband structure. As required by any multi-resonator chipless tag RFID system (see Figure 1), the two antennas are cross-polarized; in particular, the RX antenna is y-polarized (see Figure 5a), while the TX antenna is x-polarized (see Figure 5b). The final dimensions of the two antennas are summarized in Table 1.

**Figure 6:**
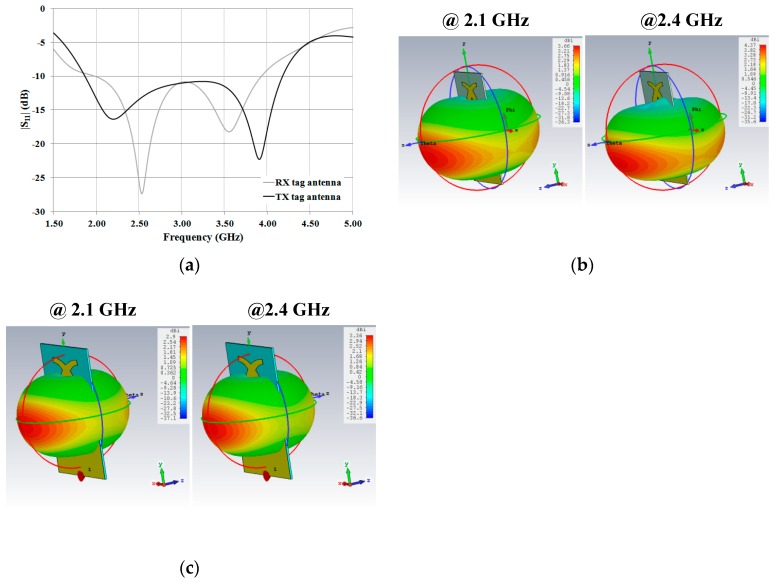
Numerical results achieved for the RX and the TX tag antennas: (**a**) comparison between the |S_11_| of the two monopoles; (**b**) 3D radiation patterns of the RX tag antenna calculated at 2.1 GHz and 2.4 GHz (b); (**c**) 3D radiation patterns of the TX antenna calculated at 2.1 GHz and 2.4 GHz.

Numerical results achieved for the reflection coefficient are reported in Figure 6a. As for the RX antenna, values of the |S_11_| lower than –10 dB were achieved in the frequency range 1.97–3.93 GHz, corresponding to a relative bandwidth of about 66.4 %. Meanwhile, the TX antenna exhibits an |S_11_| lower than –10 dB in the range 1.86–4.20 GHz, corresponding to a relative bandwidth of about 80.7 %.

As for the radiation properties, values of directivity higher than 2 dBi and a dipole-like radiation pattern were achieved in the –10 dB relative bandwidth. 3D radiation patterns calculated at 2.1 GHz and 2.4 GHz are illustrated in Figure 6b,c. Finally, all of the achieved numerical results are summarized in Table 2.

### 2.3. Overall System Tag

**Figure 7:**
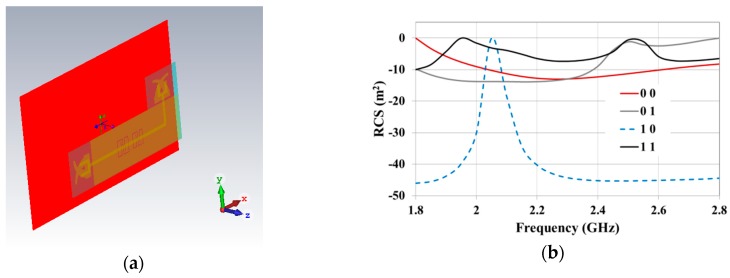
Numerical results achieved for a 2-bit overall system tag: (**a**) setup used during full-wave simulations; (**b**) numerical results achieved for the RCS of the device reported in Figure 7a.

Full-wave simulations were also performed in order to evaluate the performance of the overall tag system (i.e., the multi-stopband structure integrated with the TX and the RX tag antennas) in the case of a 2-bit configuration. The whole device is illustrated in Figure 7a. A plane wave was used as a source. According to the polarization of the RX tag antenna, the E-field was y-polarized. The results achieved for the Radar Cross Section (RCS) are illustrated in Figure 7b. In this case, a relative maximum of the RCS corresponds to a logic 1. As can be noticed, good results were obtained for the second bit, i.e., the one corresponding to the resonator having a higher frequency of resonance. In fact, for the second bit, it can be seen that the frequency at which the RCS maximum is positioned is stable (the same frequency was obtained for the configurations 11 and 01), and a difference higher than 10 dB can be observed between the RCS amplitudes corresponding to the two logical states. As per the first bit, it can be seen that the first relative maximum of the RCS is positioned at a different frequency for the configuration 11 and 10. However, also in this case, the two logical states can be easily recognized; in fact, a difference of about 10 dB was obtained for the amplitude of the RCS corresponding to the two states. 

## 3. Experimental Results

In order to verify the numerical data presented in the previous Section, some prototypes were realized and characterized. In more detail, three different prototypes were fabricated: 1) a prototype of the stop-band microstrip line in a 2-bit configuration; 2) a prototype of the TX and the RX antennas; 3) a prototype of the stopband microstrip line integrated with the RX antenna. The achieved results are described in the following. All the results refer to an implementation on a layer of pile with 0.5 mm thickness, and by using an adhesive non-woven conductive fabric for all the conductive parts.

### 3.1. Stop-Band Microstrip Line

In Reference [33] the authors presented the results referring to a stopband microstrip line in a 3-bit configuration (i.e., the configuration having three resonators slotted on the ground plane). The achieved experimental results were slightly different to the numerical ones. From the foregoing analysis, it was concluded that the mismatch between numerical and experimental results obtained for the 3-bit configuration was likely due to some imperfections of the fabrication process. In particular, the prototype presented in Reference [33] was fabricated on a 1 mm layer of pile achieved by sewing two layers, 0.5 mm thick. By analyzing the prototype, it was verified that the hand-stitching led to a non-uniform thickness of the pile. Additionally, further imperfections were introduced by the cutting plotter used for shaping the non-woven conductive fabric; in fact, the adopted plotter is more suitable for processing small areas.

Taking into account these considerations, to avoid the hand-sticking and to have a smaller area to be processed, the prototype presented in this paper is a stopband microstrip line in a 2-bit configuration fabricated on a single layer of pile (thickness 0.5 mm, dielectric permittivity equal to 1.18). As in Reference [33], an adhesive non-woven conductive fabric produced by Saint Gobain was used for all the conductive parts and a cutting plotter was used for the fabrication [9,10]. 

The final geometrical parameters of the prototype are reported in Table 3. The scale factor (see Equation (1)) was optimized for achieving a resonance shift of about 300 MHz. In particular, the two resonators were designed to introduce two stopbands centered at 2.1 GHz and 2.4 GHz.

**Figure 8:**
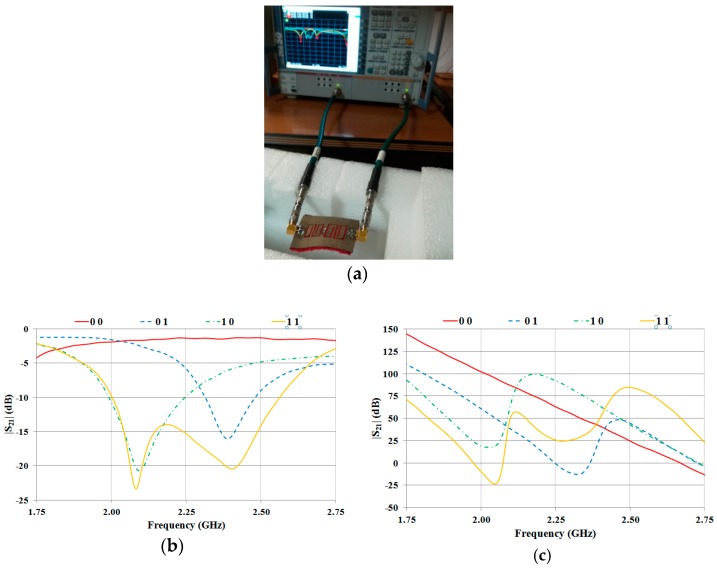
Experimental results achieved for a 2-bit prototype: (**a**) 2-bit prototype under test; (**b**) |S_21_| corresponding to all possible configurations; (**c**) phase of the S_21_ corresponding to all the possible configurations.

A picture of the 2-bit prototype is reported in Figure 8a. Experimental tests were performed through a VNA R&S ZVA50. The results for both the amplitude and the phase of the S_21_ parameter are illustrated in Figure 8b,c, respectively. A good agreement between the numerical data and experimental results was achieved. In particular, the first minimum of the |S_21_| is centered at 2.08 GHz and the second one at 2.40 GHz. Hence, with respect to the numerical data, there is a shift of about 20 MHz for the resonance corresponding to the first resonator; while the resonance of the second resonator is perfectly tuned. These results confirm that, as supposed by the authors, the disagreement between numerical data and experimental results achieved in Reference [33] was mainly due to some imperfections in the manufacturing process such as irregular thickness of the pile and cutting plotter, unsuitable to process large area.

### 3.2. TX and RX Tag Antennas

**Figure 9:**
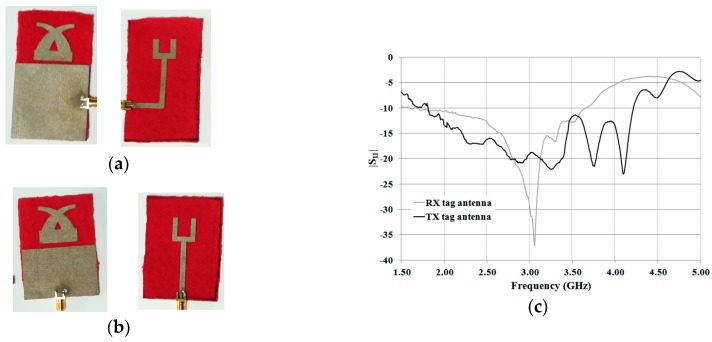
Experimental results achieved for the TX tag antenna and the RX tag antenna: (**a**) front view and back view of the RX antenna prototype; (**b**) front view and back view of the TX antenna prototype; (**c**) measured reflection coefficients.

In order to verify the numerical data of the antennas presented in the previous section, a prototype of both the TX and the RX antenna using the dimensions reported in Table 1 was fabricated (see Figure 9a,b). The experimental results achieved for the reflection coefficients are reported in Figure 9c. In both cases, a good agreement between the numerical and experimental results was obtained. From Figure 9c, it can be seen that both of the antennas exhibit values of the reflection coefficient lower than –10 dB in the frequency range of interest (i.e., 2–3 GHz).

### 3.3. 2-Bit Tag

**Figure 10:**
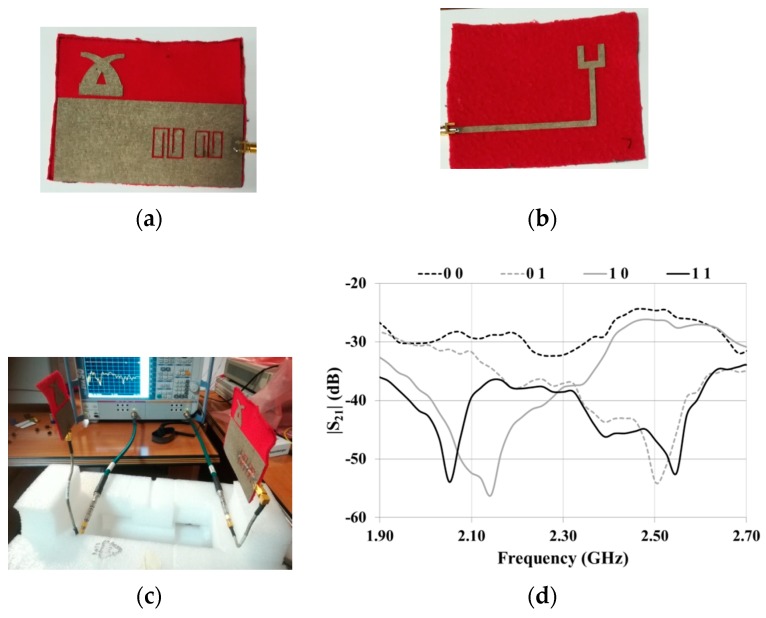
Experimental results achieved for the 2-bit tag. (**a**) Front and (**b**) back view of the stopband microstrip line integrated with the RX antenna; (**c**) experimental setup adopted for measurements; (**d**) measured transmission coefficients for the four configurations of interest.

Finally, a prototype of the stopband microstrip line integrated with the RX antenna was realized (Figure 10a,b). The overall dimensions of the prototype are 85.9 mm X 110 mm. The expected performance was verified by adopting the setup illustrated in Figure 10c. 

In more detail, two-port measurements were performed with the antenna illustrated in Figure 9b connected to Port 1 and the microstrip line integrated with the RX antenna connected to Port 2.

The transmission coefficient measured for all of the configurations of interest is reported in Figure 10d. As can be noticed, the results are very promising; in fact, even though the positions of the relative minima of the transmission coefficient are slightly different for different configurations (see, for instance, the position of the first minimum for the configurations 11 and 10), the presence of a logic 0 or a logic 1 can be easily recognized.

It would probably be possible to further improve the results by optimizing the geometry of the antennas and the transition between the stopband structure and the antenna feed-line. However, the results shown in Figure 10d fully demonstrate the feasibility of the proposed design approach and of its fully textile implementation.

## 4. Conclusions

In this paper, a fully textile frequency-signature RFID chipless tag was presented. The proposed tag encodes the desired information by exploiting a microstrip line loaded with multiple resonators slotted on the ground plane. Each resonator introduces at its frequency of resonance a relative minimum on the transmission coefficient of the microstrip line. Accordingly, by designing the resonators to have different frequencies of resonance, it is possible to encapsulate the binary code in the frequency signature of the microstrip line.

As for the antennas, the same geometry of a planar broadband monopole was applied to both the transmitting and the receiving tag antenna. 

Experimental data demonstrate the feasibility and good perspectives of the proposed design approach and of its fully textile implementation. 

## Figures and Tables

**Table 1:** Dimensions of the antennas illustrated in Figure 5.

RX antenna				TX antenna			
W_RX_	L_RX_	GND_RX_	RAD_RX_	W_TX_	L_TX_	GND_TX_	RAD_TX_
50	85.9	50	26	50	70	34.1	26

All parameters are in millimeters.

**Table 2:** Numerical results achieved for the RX and the TX tag antennas.

	BW_-10 dB_ (GHz)	|S_11_| (dB)			Directivity (dBi)		
		@ 2 GHz	@ 3 GHz	@ 4 GHz	@ 2 GHz	@ 3 GHz	@ 4 GHz
**RX**	1.97–3.93	–10.1	–10.9	–9.14	3.40	3.99	3.44
**TX**	1.86–4.2	–13.3	–11.03	–17.9	2.75	4.06	4.17

**Table 3:** Geometrical parameters of the 2-bit prototype shown in Figure 8a.

2-bits Prototype								
W	L	W_feed_	W_res_	L_res_	t_res_	d_res_	g_res_	S_F2_
40	60	3.7	19.1	18.3	1.2	2	4	0.77

All parameters are in millimeters.
